# Compact 3D-Printed Unit for Separation of Simple Gas Mixtures Combined with Chemiresistive Sensors

**DOI:** 10.3390/s24134391

**Published:** 2024-07-06

**Authors:** Magdalena Zvonkova, Martin Adamek, Nela Skowronkova, Stepan Dlabaja, Jiri Matyas, Miroslav Jasso, Anna Adamkova, Jiri Mlcek, Richardos Nikolaos Salek, Martin Buran

**Affiliations:** 1 Department of Food Analysis and Chemistry, Faculty of Technology, Tomas Bata University in Zlin, Vavreckova 5669, 760 01 Zlin, Czech Republic; m1_zvonkova@utb.cz (M.Z.); n_skowronkova@utb.cz (N.S.); m_jasso@utb.cz (M.J.); 2 Department of Automation and Control Engineering, Faculty of Applied Informatics, Tomas Bata University in Zlin, Nad Stranemi 4511, 760 05 Zlin, Czech Republic; m2adamek@utb.cz (M.A.); sdlabaja@utb.cz (S.D.); 3 Centre of Polymer Systems, University Institute, Tomas Bata University in Zlin, Trida Tomase Bati 5678, 760 01 Zlin, Czech Republic; matyas@utb.cz; 4 Department of Food Technology, Faculty of Technology, Tomas Bata University in Zlin, Vavreckova 5669, 760 01 Zlin, Czech Republic; rsalek@utb.cz; 5 Department of Microelectronics, Faculty of Electrical Engineering and Communication, Brno University of Technology, Technicka 3058/10, 616 00 Brno, Czech Republic; martin.buran@vutbr.cz

**Keywords:** chemiresistive gas sensors, 3D printing, polylactic acid, capillary, sustainability

## Abstract

Inexpensive chemiresistive sensors are often insufficiently selective as they are sensitive to multiple components of the gas mixture at the same time. One solution would be to insert a device in front of the sensor that separates the measured gas mixture and possibly isolates the unwanted components. This study focused on the fabrication and characterization of a compact unit, which was fabricated by 3D printing, for the separation and detection of simple gas mixtures. The capillary, the basic part of the compact unit, was 4.689 m long and had a diameter of 0.7 mm. The compact unit also contained a mixing chamber on the inlet side and a measuring chamber with a MiCS-6814 sensor on the outlet side. Mixtures of ethanol and water at different concentrations were chosen for characterization. The measured calibration curve was found to have a reliability of R^2^ = 0.9941. The study further addressed the elements of environmental friendliness of the materials used and their sustainability.

## 1. Introduction

Although there are still challenges to overcome, 3D printing has become a favored approach currently employed in the field of gas sensor analysis [1]. One of the biggest advantages of diverse 3D printing techniques is their modifiability based on potential applications and uses; thus, 3D printing of gas sensors and various components connected to them paves the way for further research while offering a wider range of applicability [2,3]. As described by Zhou et al. [2], 3D printing can be employed for the manufacturing of additional components including the cells, channels, circuits, electrodes, chambers used for diverse purposes such as gas preprocessing or the improvement in sensors’ sensing performance [2]. The structural components involved in the construction of the final device can also be 3D printed, as reviewed by the aforementioned authors. They can function not only as customized holders for commercial sensors [4] but also as protection for the sensors from moisture [5] or dust [6].

This study focuses on the idea of using a simple capillary chromatographic column made from biodegradable materials via common 3D printing for the separation of a sample into individual components before their measurement using low-cost chemiresistive sensors. The reason for doing this pertains to the sensitivity of these sensors to several gases simultaneously. Without additional devices or information about the sample, a single sensor (sensory element) cannot provide detailed information about the unknown sample being measured. It can only determine a response to the entire group of gases specified by the manufacturer [7]. Therefore, the improvements described further in this article are aimed at modifying the resolution capability of the system and developing a device that would not only be able to separate and subsequently detect components of simple gas mixtures, but also possibly semi-quantify different analytes. The aim was to incorporate not only the capillary column but also other parts of the measuring chain, including the mixing chamber and the detector’s measuring chamber, into one compact unit. This is one of the key aspects characterizing the novelty of this study.

With both the aforementioned objectives in mind, great emphasis was also placed on finding a sustainable solution, i.e., all the chosen components of the manufactured device were either biodegradable or recyclable. Therefore, polylactic acid (PLA) was chosen for the 3D printing of the capillary and other components, such as the holders and the mixing chamber. In the global search for sustainable materials, PLA has emerged as a suitable alternative to other polymers in selected applications, mainly because of its biodegradability, availability, and convenient mechanical and thermal properties [8]. Furthermore, lactic acid (LA) monomers can be obtained from renewable sources such as potato, sugarcane or corn by microbial fermentation, and then PLA can be produced using a simple, cost- and energy-efficient synthesis [9]. Even though PLA synthesis is considered energy-efficient, the most demanding process in terms of CO_2_ releasing into the atmosphere is the actual conversion of the agricultural products to PLA. By the optimization of the synthesis reactions, PLA could also become a low-carbon material [10]. Currently, there are several PLA degradation strategies, such as landfilling, composting, anaerobic digestion, incineration or thermal treatment, and chemical and mechanical recycling [11]. The environmental footprint of PLA’s degradation can be minimized by chemical (molecular) recycling—depolymerization—and the repeated use of LA monomers to synthesize new material [12]. Regarding energy consumption, this approach is preferable to the repeated production of LA from starchy agricultural waste [13]. Researchers have also suggested the possibility of the direct reprocessing of 3D-printed PLA products back into the filaments used for 3D printing without significant degradation of the material [14].

In addition to the aforementioned components made from PLA, other materials used for the construction of the device included plastic, cardboard, and metal, which are fully recyclable or reprocessable in the Czech Republic. Plastic syringes served as a dispensing device for the carrier gas, while metal parts were employed to ensure the mechanical functionality of the device, and all the components were subsequently mounted on a cardboard box, which provided the basic structural backbone of the whole setup.

The only non-biodegradable or non-recyclable parts used were the electronic components such as electrical cables or gas sensors, which are reusable and can either be removed and used in other experimental configurations or replaced, if necessary, and the remaining parts of the device can be used until any mechanical defects occur. In general, the operating lifetime of the sensors varies according to the frequency of use or the gas concentrations that are applied to the sensors, but it is also based on their storage and usage conditions such as temperature or humidity [15,16,17].

## 2. Materials and Methods

The principle of the entire device is based on the idea of utilizing a simple capillary chromatographic column manufactured by 3D printing for the separation of samples into components prior to their measurement using inexpensive chemiresistive sensors.

The sample is dispensed from a syringe into a stream of clean air flowing through the capillary column. Within this column, the mixture is separated into individual components that arrive at the detector (chemiresistive sensor) at different times. The detector is housed in a closed chamber at the end of the column, and upon the arrival of molecular components from the sample, it reacts by reducing the electrical resistance of the sensitive layer. This decrease in electrical resistance would be recorded as a decrease in electrical voltage at the specific sensitive element and then converted into an equivalent digital value by an A/D converter, which would then be recorded and further processed by a microcontroller. From the values obtained from the A/D converter, it was possible to determine the ratio of the resistance of the sensitive layer during analyte detection to the resistance when the sensitive layer is placed in clean air. From this ratio, the concentration of the analyte could be calculated. However, this applies only under the condition that the monitored gas is known and that there are no other gases in the observed gas mixture to which the sensor reacts. These conditions cannot be met by unknown analytes. Therefore, a separation capillary chromatographic column was used in the study for sample processing.

### 2.1. Design and Development of the Capillary and the Device

The measuring device consists of several interconnected blocks. The core block of the measuring device is a compact unit made by 3D printing technology, integrating a capillary for the separation of the components of the mixture, a mixing chamber for mixing the gas sample with the carrier gas, and a measuring chamber equipped with a chemiresistive sensor. The apparatus further includes a sample dispensing device and a carrier gas dispensing device with a motor and a gearbox. Everything is controlled and scanned by a control and measurement unit, which is controlled by an ESP-32 microcontroller.

**Figure 1:**
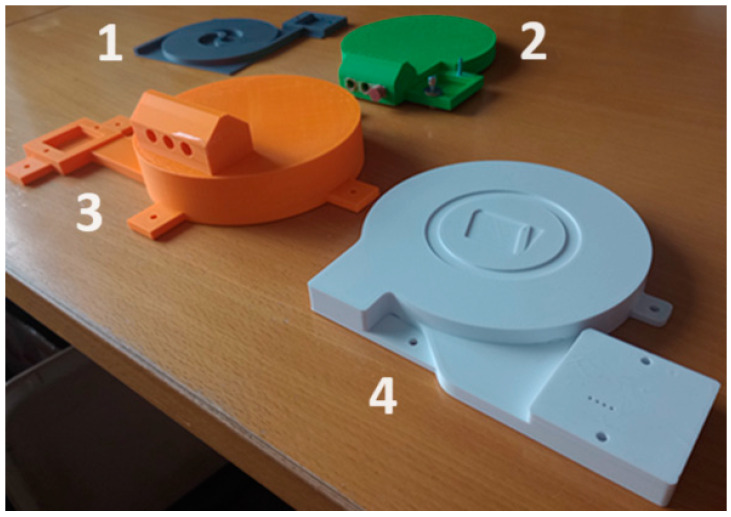
Different capillary blocks with mixing and measuring chamber during development. The grey block (1) is the original prototype from the previous study by Adámek et al. [7], the green (2) and orange (3) blocks are improved prototypes, and the white block (4) is used within this article.

The main part is a compact block with a capillary, the design of which was based on earlier research described in Adámek et al. [7]. As part of the development, several various types of these blocks had been designed and printed (Figure 1) during the study. The grey block (1) represents the original prototype from the previous study by Adámek et al. [7], while the green (2) and orange (3) blocks are prototypes of the improved type. The white block (4) is utilized within this paper.

**Figure 2:**
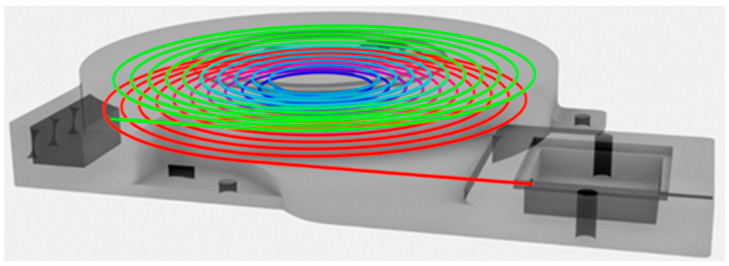
Internal arrangement of the compact block comprising of two mirrored helices.

**Figure 3:**
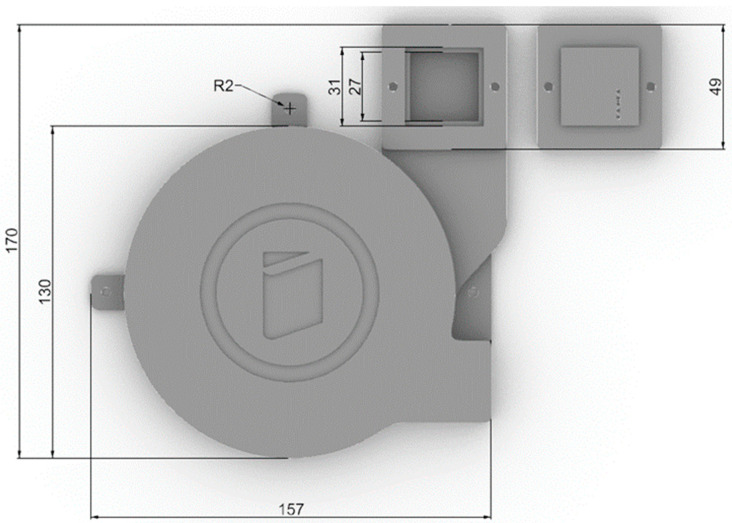
External dimensions of the compact block and square measuring chamber.

Emphasis was placed on the appropriate integration of the mixing and measuring chambers into a compact unit with the capillary (Figure 2 and Figure 3). The diameter of the capillary was reduced to d = 0.7 mm. Overall, the capillary diameter was reduced by nearly 42% compared to the model presented in the previous paper [7].

The two-tier capillary’s length was 4.689 m, which is comparable to the previously described three-tier capillary with a length of 4.692 m. Its design featured two mirrored helices interconnected by a continuous curve, ensuring that there were no excessive fractures that could hinder the printing process. The capillary was successfully printed using a commercially available Bambu Lab PS1 printer (Bambu Lab, Shenzhen, China) equipped with a standard 0.4 mm nozzle. Figure 3 illustrates the external dimensions of the compact block and the square measuring chamber.

**Figure 4:**
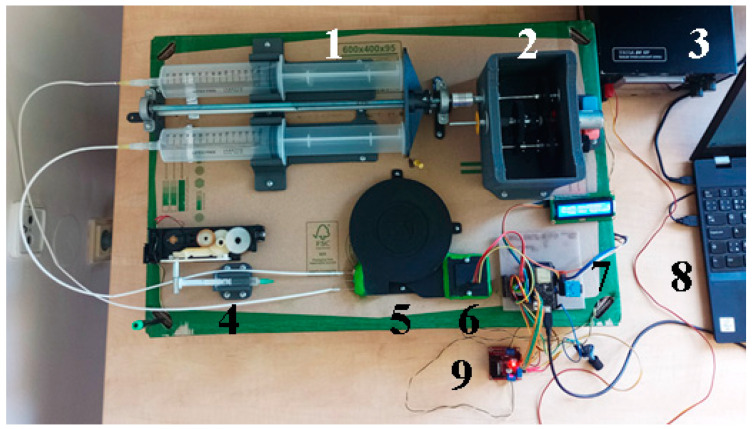
Complete real arrangement of the experimental measuring system (1—syringes for the carrier gas, 2—two-speed gearbox, 3—power supply for motors, 4—sample dispenser, 5—capillary block, 6—measuring chamber, 7—control board with ESP-32 microcontroller, 8—computer for data acquisition, 9—motor driver).

Sample and carrier air dispensers were also positioned on the recyclable paper substrate (Figure 4). The principle and basic use were the same as the system described in the previous article [7]. The 2 mL syringe holders for the sample and the two 150 mL syringes for the carrier gas were new (pure air was used; the full syringe volume is 165 mL). Both the carrier gas and sample were drawn into syringes by the equipment operator. Normal room air served as the carrier gas, filtered through a NY 0.22 µm syringe filter (Chromservis s.r.o., Prague, Czech Republic). Immediately before gas measurement, a sample was drawn into the syringe through the needle, positioning the tip just above or lightly touching the liquid surface, ensuring the needle lumen mouth was not submerged in the liquid. The syringe containing the sample was then inserted into the measuring system, and the measurement commenced. The carrier gas was expelled from the syringes by a pushing mechanism with a newly designed construction and a gearbox. This allowed the pushing mechanism to move forward and backward, and it was adapted to work in slow and fast modes during repositioning. The mode change was possible when the operator mechanically adjusted the position of one of the gear wheels. The new syringe holders and new pushing mechanism ensured better stability of the syringe position, higher stability of the output volume rate, and reduced risk of the syringe piston stalling or crossing. The basic parts of the system were 3D printed from PLA material, increasing the portability of the system (weight reduction) and increasing its recyclability.

**Figure 5:**
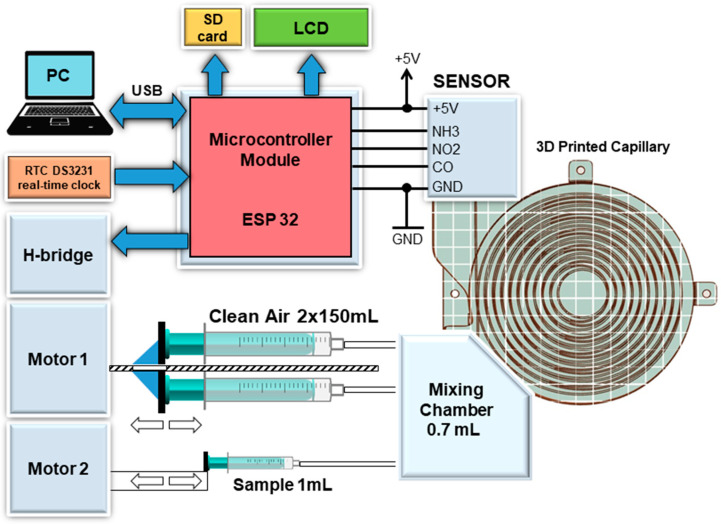
Experimental measuring system—Connection of main components.

The last part, mounted on the substrate, is the control and measurement unit (Figure 5). The central part of this section is the ESP-WROM-32 microcontroller (Espressif Systems, Shanghai, China) on the board Wemos LoLin 32 ESP-WROOM-32, controlling the user interface (display, pushbutton), operating the H-bridges for motor control based on the L298N circuit, sensing signals from the MiCS-6814 sensor (SGX Sensortech, Neuchâtel, Switzerland) and communicating with a PC-class computer using a common USB/COM interface with a communication speed of 115,200 Bd.

The MiCS-6814 is a compact MOS sensor containing three fully independent sensing elements (RED, OX and NH3) on one package [18]. The microcontroller is also connected to the RTC DS 3231 real-time clock and SD memory card. This solution allowed the device to work without a connected computer and computer communication infrastructure and allowed for easier data transfer and backup. In addition to higher processing power, greater communication capabilities, and the ability to store more data, one of the main advantages of this microcontroller was the higher resolution of the A/D converter (12 b instead of the original 10 b). This allowed for a further increase in accuracy of the sensed signal.

### 2.2. Measurement Methodology

The measurement methodology was similar to the one described in [7] and modified as follows. After starting the measurement, the mixing chamber, the capillary, and the measuring chamber undergo cleaning with the carrier gas (clean air) for a duration of t = 30 s. Subsequently, the sample is injected into the measuring chamber, and the carrier gas is gradually displaced from the syringes at a constant rate. The measurement is terminated once the carrier gas is fully expelled from the syringes.

Upon completion of the sample measurement, the system is flushed with 330 mL of carrier gas (the measurement is performed without the injection of the sample). The rate of carrier gas displacement and consequently the total measurement time are controlled by the motor voltage.

For instance, with a slow gearbox speed and a voltage of U = 12 V at the motor, along with a full carrier gas syringe volume set, the total gas displacement time amounts to t = 830 s, achieving a gas displacement rate of Q = 0.397 mL/s. When utilizing a fast gearbox speed and a voltage of U = 6 V on the motor, alongside a full carrier gas syringe volume set, the total gas displacement time is reduced to t = 240 s, resulting in a higher gas displacement rate of Q = 1375 mL/s. This configuration was employed for the measurements. In the final part, the piston was able to push against the syringe wall, therefore only the first 235 samples were considered in the processing.

Notably, 96% ethanol (Ing. Petr Švec-PENTA s.r.o., Prague, Czech Republic, CAS: 64-17-5, EINECS: 200-578-6) was used as a base material for preparing the measuring solutions. From the stock solution, solutions of 6%, 12%, 24%, and 48% were mixed by sequential dilution. Demineralized water was used for the dilution. Sampling from the sample bottle was performed using a needle syringe, with the needle positioned just above the sample level.

### 2.3. Evaluation Methodology

The evaluation methodology mirrors the one outlined in the paper by Adámek et al. [7], with a slight modification concerning the increase in the maximum number of output signal levels from the A/D converter. Specifically, the range extended from 0 to 4095 instead of the previous range from 0 to 1023, accommodating smaller input voltage variations within the range of 0 to 3.3 V as opposed to 0 to 5 V. Notably, these adjustments were compared following normalization, relative to the initiation of the measurement at time t = 30 s.

For data processing and graphical representation of the measurement outcomes, Microsoft Excel 2019 (Microsoft Corporation, Redmond, WA, USA) was used.

## 3. Results and Discussion

### 3.1. Reproducibility of the Measurements

Pure air was measured as a first set of samples, serving as a carrier gas. These results were used to determine the background values for the unsampled measurements and further used for standardization in order to eliminate the background effect caused by the carrier gas. Successive measurements of ethanol samples of 6%, 12%, 24%, 48%, and 96% concentration were then taken.

**Figure 6:**
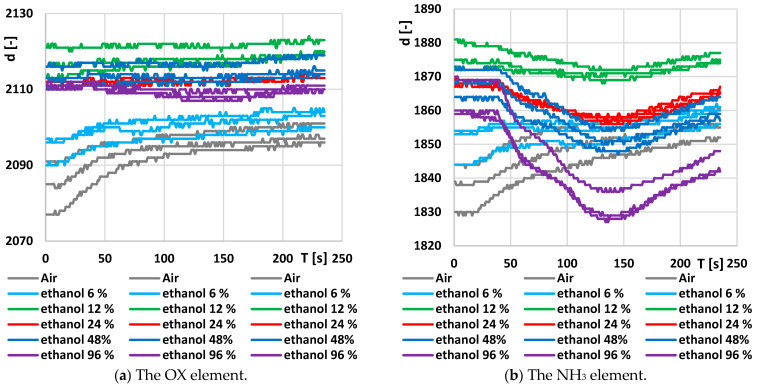
Original output time response on the output of the A/D converter connected to the individual elements of the combined sensor MiCS-6814: (**a**) The OX element; (**b**) The NH_3_ element.

Since the OX element in the combined sensor MiCS-6814 reacts mainly to NO_2_ and NO, and it does not respond to ethanol, the results from this sensor will only be briefly described. Emphasis will instead be placed on the RED element, which is the most reactive part of MiCS-6814 to ethanol. The original responses from the OX and NH_3_ elements are shown in Figure 6. While changes in the NH_3_ element’s dependence on ethanol concentration can be observed, the post-processed signals have proven unsuitable for more precise analysis.

**Figure 7:**
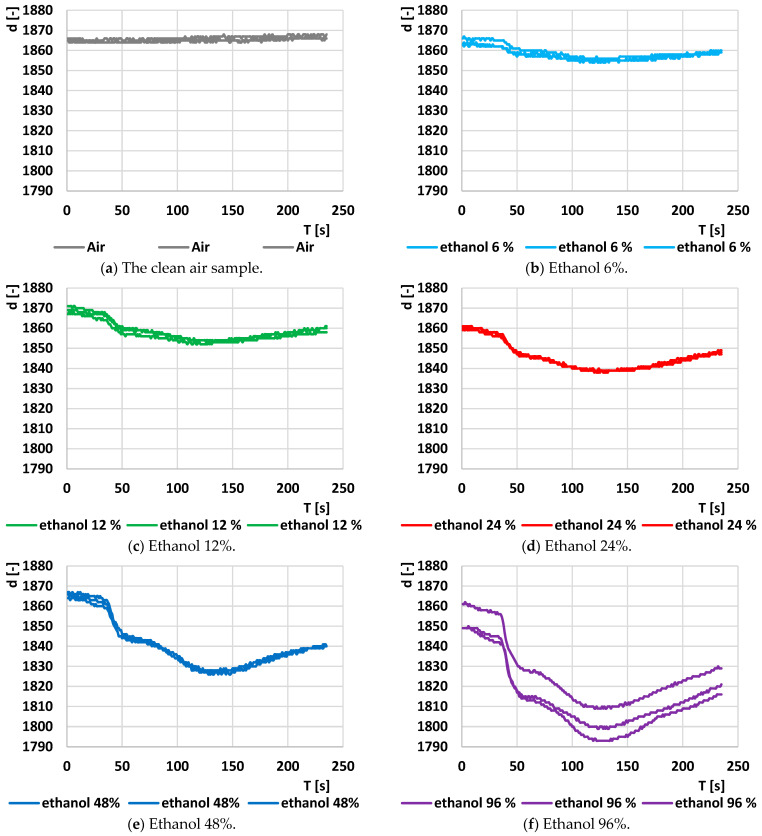
Original output time response at the A/D converter output (d [-]) linked to the RED element of the combined sensor MiCS-6814 at various ethanol concentrations. The *Y*-axis scale (d [-]) is consistent across all plots to facilitate comparison of measured values’ reproducibility.

The RED element exhibited the strongest and most consistent responses to the samples with varying ethanol concentrations. The responses to each sample with different ethanol concentrations are depicted in Figure 7. The *Y*-axis scale (d [-]) remains consistent across all the plots to facilitate the comparison of measurement reproducibility. Apart from the sample with 96% ethanol, the response curves are relatively close to each other, indicating good reproducibility. This is further supported by the maximum values of standard deviation calculated from the measured values of the three characteristics at the specified ethanol concentration and time, as shown in Table 1.

Table 1 also indicates the maximum difference between these characteristics at a consistent ethanol concentration and time. It shows that a value of 3 steps per A/D converter (approximately 2.5 mV) represents the maximum difference between the measured curves for air, which can be considered as the maximum noise level. This value is comparable to the values observed for the other measured sets of characteristics across different ethanol concentrations (2–5 steps). The sampling standard deviation is less than 3 A/D converter steps for all concentrations (lower than the difference between the measured curves for pure air). Therefore, the reproducibility of the measurements can be considered adequate. The exception is observed with 96% ethanol, where a maximum sampling standard deviation of 8.62 steps is determined.

### 3.2. Standardization of Data

**Figure 8:**
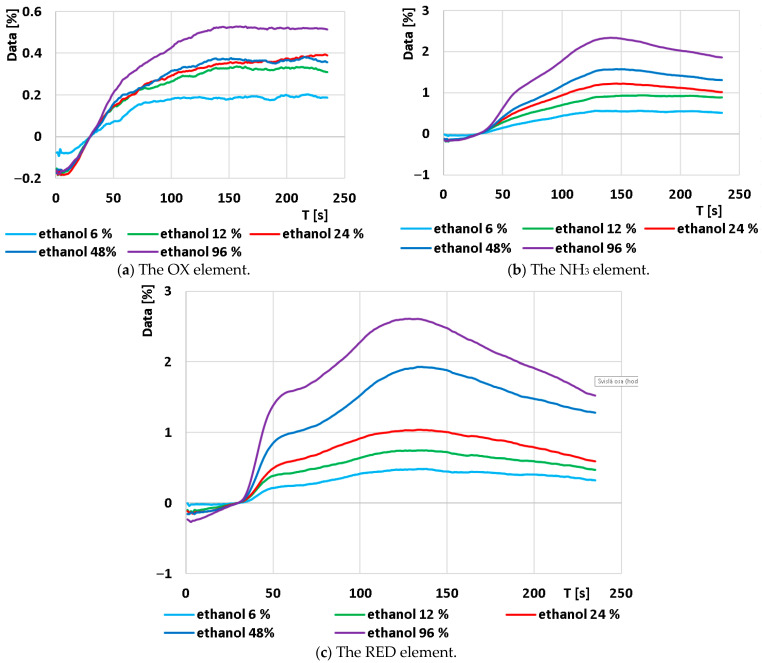
Average output time response of each element after standardization for each concentration set: (**a**) The OX element; (**b**) the NH_3_ element; and (**c**) the RED element.

The next step in data processing involved averaging each set of curves for every ethanol concentration into a single curve representing the average value. Subsequently, these average curves were smoothed using a mean moving average (m = 11) and then standardized according to [7]. A larger moving average range resulted in smoother curves and reduced noise. However, smoothing in this manner increased the likelihood of obscuring small yet significant changes on the curve that may be considered important. The resulting graphs for the individual sensors are displayed in Figure 8.

The graph of the OX element exhibits noticeable waviness in the curves, small amplitude changes, and an uneven distribution of the curves. Consequently, the data processing from this element is unsuitable for analytical purposes in this type of measurement.

In contrast, the NH_3_ element shows a well-distributed magnitude of change in the curves, but as will be detailed below, the curves are consistently under-smoothed.

The RED element demonstrates the strongest response signal, consistent with the manufacturer’s claim of highest sensitivity to ethanol. The signals are therefore adequate for further processing, appropriately dispersed, and the curves appear sufficiently smoothed.

### 3.3. Ethanol Calibration Curve

**Figure 9:**
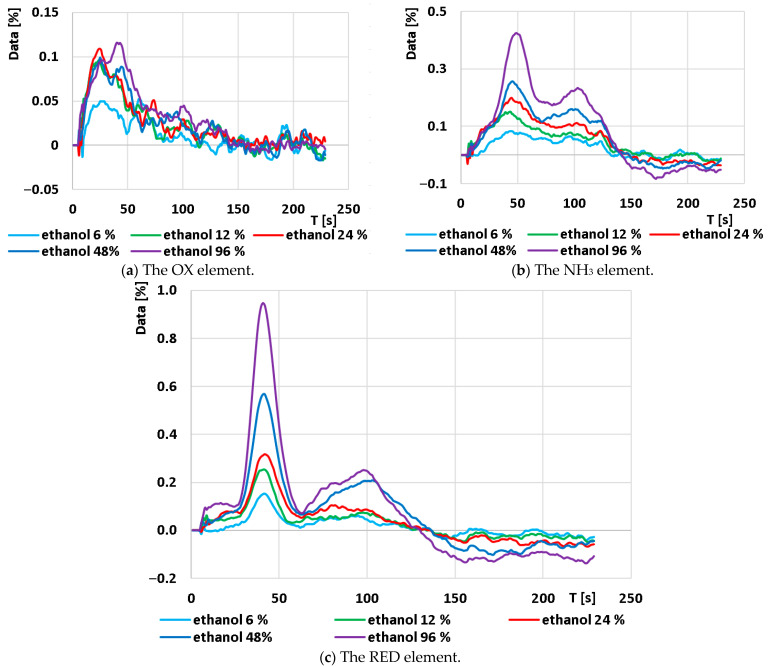
Time response of each element after mean difference calculation for each set of concentrations: (**a**) The OX element; (**b**) the NH_3_ element; and (**c**) the RED element.

The last operation was the mean difference calculation. In order to follow the same procedure with the measurements provided in the previously published paper [7], m = 11 was chosen with the calculation Δd_x_ = d_x+5_ − d_x−5_. The resulting characteristics are shown in Figure 9.

As mentioned above, the signal from the OX element was not suitable for analysis in this case. The signal from the NH_3_ element was not suitable for full-scale analytical processing because the time of peak maxima was shifted and, for some curves, the maximum peak value could not be accurately determined. The separation of the gas mixture into two components (probably ethanol and water vapor) can be seen from the graph. This demonstrates that the designed capillary allowed for the separation of the mixture into its individual components. This can be seen more clearly in the characteristics obtained from the RED element. At time t = 41 s, a clear peak is visible in the patterns, which is suitable for characterization. Approximately 100 s after this peak, another peak follows, but the position of the peak is no longer clear and is therefore not suitable for analysis. However, this peak again shows that the capillary allowed the mixture to be separated. This shows the correct function of the capillary and confirms the assumptions about the possibility of separating the gas mixture prior to measurement on a low-cost chemiresistive sensor using a 3D-printed capillary.

As the described design modifications were made to the device, compared to the previous study [7], this experiment was able to validate the data obtained in the previous pilot experiments and therefore the further use of this system for the detection of a wider range of analytes can be considered. Similar experiments using a 3D-printed column with gas sensors and the development of a simple gas chromatograph were described by Zaidi et al. [19,20], who successfully used the device to separate single gas mixtures. Their experiments focused on the use of a pre-separation 3D-printed column for the detection of ethylene and humidity in ripening bananas [19,20]. In their latest study in this field, they developed a ceramic preconcentrator that enhanced the system’s resolution and ensured reliable measurement repeatability. The importance of these straightforward devices for applications in the food industry was also highlighted [21].

Comparing our data with other authors may be challenging; although they describe the combination of gas sensors with column systems, the devices are often much more complex than those presented in our experiments, e.g., using specialized columns for micro gas chromatography. Despite using an advanced µGC column containing Tenax TA particles, the study presented by van der Broek et al. [22] provided an interesting insight into the quantification of methanol in a mixture with an abundant ethanol concentration. Their experiment on the detection of analytes from the same chemical family could be the basis for further tests performed by our proposed experimental setup. One future possibility is to employ the device to detect other simple volatile compounds such as methanol. Methanol content in spirits represents one of the most serious health threats of adulterated alcoholic beverages [23] because it might lead to permanent visual damage and even death [24]. Detecting methanol content in alcoholic beverage samples is of paramount importance, as the data collected during three documented mass methanol poisonings in 2001 (Estonia), 2002 (Norway) and 2012 (Czech Republic) indicate that the mortality rate of these events ranged between 29 and 36% [25,26]. A simple, inexpensive, and portable device for the accurate detection of methanol could therefore help prevent these incidents. First, it will be necessary to test whether the MiCS-6814 sensor can also detect methanol and possibly distinguish it from chemically similar ethanol. Should this experiment be inconclusive, the next option would be to consider replacing the sensor with one capable of detecting both substances.

The other promising approach is to detect the adulteration of alcoholic beverages by diluting them with water, which is one of the easiest ways to adulterate alcoholic beverages [27]. The concentration of ethanol could be easily determined using the obtained ethanol calibration curve and afterwards compared to the concentration stated by the producer on the label. While this type of adulteration generally poses no risk to the consumer, it is considered to be an unfair practice, which is misleading to potential consumers.

**Figure 10:**
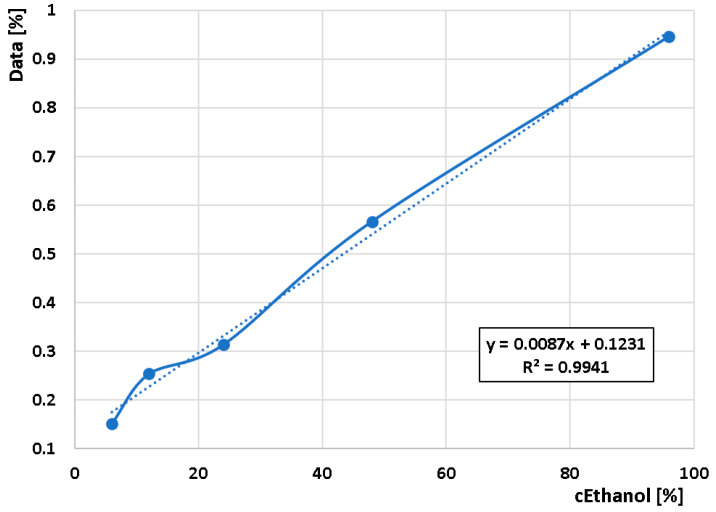
Ethanol calibration curve for a 3D-printed PLA capillary combined with MiCS-6814 chemiresistive sensor.

Since the first peak in the characteristics is assumed to be for ethanol and the second for water vapor, a calibration curve was generated at time t = 41 s for the maximum values of the first peak in the characteristics measured with the RED element. The calibration curve is shown in Figure 10. The curve has a reliability of R^2^ = 0.9941, which, although considered inferior in conventional analytical methods of chemical analysis, can be considered very successful in this simple experimental setup.

The study created a device that is unique in its nature. Not only does this device integrate a mixing chamber, a separation chromatographic column, and a measuring chamber for the detector into a single unit, but it is also manufactured with a standard 3D printer using commonly available biodegradable PLA material accessible to the general public. Unfortunately, this somewhat complicates comparisons of this device with literary sources because most authors produce 3D capillaries for other chemical methods (most commonly capillary electrophoresis) [28,29,30], different types of chromatography (liquid) [31,32], from different materials (eq. metal) [32,33], or with different types of 3D printing, typically more expensive (microfluidic chip capillaries or SLA printing) [34,35,36]. For these reasons, devices from other authors have different parameters, which are challenging to discuss globally with the parameters of the device presented in the study. Thus, the study fills an information gap in this area and brings new insights to science and research.

## 4. Conclusions

This study addresses the sensitivity problem of inexpensive chemiresistive sensors to multiple components in a gas mixture. To separate the mixture, a compact unit was fabricated by 3D printing, containing a mixing chamber, a 4.689 m long capillary with a diameter of 0.7 mm, and a measuring chamber. The designed and fabricated compact unit was demonstrated to be functional, where the capillary was able to separate the gas mixture sample. This verified the results obtained in the previous studies. A calibration curve was determined for the test mixture composed of different concentrations of ethanol and deionized water. The calibration’s curve reliability was found to be R^2^ = 0.9941, which can be considered a successful value for this experimental device. 

The study demonstrated the feasibility of fabricating a low-cost gas separation device from a sustainable material (PLA, paper, metal) that could be used as a pre-separation column prior to measurements with chemiresistive sensors. Further development of the device mentioned in previous studies enabled the increase in the sensors’ resolution, reduced the cost, and expanded their capabilities. The study is therefore one of the next steps towards the production of a compact 3D-printed portable chromatograph.

## Figures and Tables

**Table 1:** Maximum standard deviation and maximum difference values for each characteristic set across different ethanol concentrations in the sample.

Ethanol Concentration	0%	6%	12%	24%	48%	96%
Maximum SD	1.73	2.52	2.08	1.15	2.52	8.62
Maximum d_max_ − d_min_	3	5	4	2	5	17

## Data Availability

New research data were presented in this contribution.
